# Bacteremia following dental implant surgery: Preliminary results

**DOI:** 10.4317/medoral.17263

**Published:** 2011-12-06

**Authors:** Nilüfer Bölükbaşı, Tayfun Özdemir, Lütfiye Öksüz, Nezahat Gürler

**Affiliations:** 1 Dr. PhD, Department of Oral Implantology, Faculty of Dentistry, Istanbul University; 2 Dr. PhD, Professor, Department of Oral Implantology, Faculty of Dentistry, Istanbul University; 3 Dr. PhD, Department of Microbiology and Clinical Microbiology, Medical Faculty, Istanbul University; 4 Dr. PhD, Professor, Department of Microbiology and Clinical Microbiology, Medical Faculty, Istanbul University. President of Turkish Society of Microbiology

## Abstract

Objectives: The aims of this study were to investigate the incidence of bacteremia, bacteriology and antibiotic susceptibility against to causative bacteria associated with dental implant installation. Study Design: 30 generally healthy patients were enrolled in this study. Blood samples were collected at baseline and at 30 minutes after dental implant installation and 24 hours after dental implant surgery. Blood samples were cultured in a BACTEC system. The isolated bacteria were identified using conventional methods. Antimicrobial sensitivity tests were performed by disc diffusion. Results: No bacteria were isolated at the baseline and 24 hours after surgery, whereas the prevalence of bacteremia at 30 minutes after dental implant installation was 23%. The isolated bacteria species were Staphylococcus epidermidis, Eubacterium spp., Corynebacterium spp. and Streptococcus viridans. The Staphylococcus epidermidis, which was isolated in three patients, was found to be resistant to penicillin which is first choice of many clinicians. Conclusion: Our findings suggest that installation of dental implants can produce bacteremia. Within the limitations of this study, it can be speculated that the resistance of antibiotics may compromise the routine prophylaxis against infective endocarditis. Therefore use of blood cultures and antibiograms may be suggested in risky patients. The outcome of the present study should be verified using a larger patient group with varying conditions.

** Key words:** Dental implant, bacteremia, infective endocarditis, antibiotic prophylaxis.

## Introduction

The bloodstream is sterile under normal conditions. Transient bacteremia occurs when bacteria enter the bloodstream. Transient bacteremia is unavoidable. Bacteria species; general health care of the patient; and type of dental procedures are effective on the emergence of bacteremia complications (1). Bacteremia in dentistry frequently occurs following not only invasive procedures, such as extractions and periodontal surgery (2,3), but also following non-invasive procedures, such as, periodontal probing (4,5), root canal treatment (6), orthodontic treatment (7) and oral hygiene procedures (4,8,9). In a healthy person, bacteremia in the bloodstream is countered by normal defense mechanisms (4). However; bacteremia may cause infective endocarditis (IE) in patients with cardiac anomalies or in patients with a compromised immune system (10). IE is an infection of the endocardium. Valvular damage following rheumatic fever, previous endocarditis, a ventricular septal defect, prosthetic heart valves, or valvular stenosis can lead to changes in blood flow or damage in the cardiac endothelium (11). Changes in blood flow and/or damaged endothelium surfaces lead to precipitation of platelets and fibrin (11). If the bacteria enter the blood circulation, they can be colonized in the platelet and fibrin network. The network of platelet, fibrin, inflammatory cells and enclosed organisms is called "infective vegetations" (11). These vegetations can result in local myocardial abscesses, which inhibit the valvular function and, eventually, may lead to congestive heart failure. Furthermore, separating vegetations can reach distant tissues and cause damage in organs such as the brain, lungs, kidneys and spleen (11). A wide range of pathogens cause bacteremia. The most common causative bacteria are streptococci, staphylococci and enterobacteria (11). IE is difficult to diagnose, may require prolonged treatment and may be fatal if untreated. Therefore, antibiotic prophylaxis is recommended for the prevention of bacteremia in susceptible patients. Antibiotic prophylaxis aims to reduce the amount of bacteria in blood and bacteria adherence in sterile vegetations (12). 

Different scientific organizations have recommended various prophylactic antibiotic regimens. The guidelines suggested by the American Heart Association (AHA) and British Society of Antimicrobial Chemotherapy (BSAC) are often used (13-15). AHA and BSAC suggest prophylaxis in all procedures involving dentogingival manipulation or endodontics (14,15).

 Unfortunately, there is insufficient scientific data concerning the incidence of bacteremia and types of bacteria species following the installation of dental implants (16). The techniques used for the detection of bacteremia and antibiotic susceptibility tests require time to complete. Therefore, antibiotic prophylaxis for this type of surgery is performed empirically or according to AHA or BSAC recommendations.

The aims of this study were: 

- To investigate the prevalence of bacteremia related to dental implant surgery.

- To identify the microorganisms isolated from blood cultures.

- To analyze the antibiotic susceptibilities of the detected bacteria and to give information about antibiotic prophylaxis to be applied in patients at risk of IE who are planned to undergo a dental implant surgery.

## Material and Methods

 Ethical Approval

This study was approved by the Ethical Committee of Istanbul University in accordance with the World Medical Association Declaration of Helsinki (version VI, 2002 http://www.wma.net/e/policy/b3.htm). Written consent was obtained from all participants prior to the study.

 Patient Selection

The present study was carried out in the Clinic of the Department of Oral Implantology at the Faculty of Dentistry, Istanbul University. The study group comprised 30 volunteers (13 males, 17 females, mean age 41±13 years) selected from patients who meet the inclusion criteria. All participants were scheduled to undergo a maximum of 2 implants, placed without using advanced surgical techniques (such as sinus lifting or guided bone regeneration procedures). A total of 41 dental implants were placed between 2006 and 2008. The following exclusion criteria were applied: age under 18 years, systematic disease, smoking habit, any type of immunodeficiency, systematic use of antibiotics in the 3 months prior to the study, routine use of oral antiseptics, presence of odontogenic infection (e.g. aggressive periodontitis, periapical diseases and pericoronitis), and risk of IE.

 Surgical Procedure

All surgical procedures were carried out under infiltrative local anesthesia by injection of articaine hydrochloride with epinephrine (each 2 ml ampoule includes 80 mg articaine hydrochloride and 0.020 mg epinephrine) (Ultracain DS Fort, Sanofi Avantis, İstanbul, Turkey). Approximately 2 ml anesthetic solution was applied to each implant site. A full thickness flap was elevated in all surgeries. Vertical incisions were avoided as much as possible and, when necessary, were performed in a maximum one tooth away from the area in which an implant is to be positioned. Osteotomy and implant installation were performed according to the manufacturer’s surgical protocol. Wound closure was completed using silk interrupted sutures (Dogsan Medical Supplies Industry, Trabzon, Turkey). The sutures were removed one week after surgery.

 Chemotherapeutic Treatment

No preoperative or postoperative antimicrobial drugs and oral antiseptics were administered. All patients received a standard prescription for anti-inflammatories (Meloxicam, Nobel, Istanbul, Turkey) that was administered for four days, starting from the day of operation.

 Blood Sampling

To determine the bacteremia, blood samples were collected from patients at baseline (before local anesthetic injection, first sample), 30 minutes after dental implant installation (second sample) and 24 hours after the surgery (third sample). Prior to sampling, the skin was wiped with 70% isopropyl alcohol (ADR, Advanced Diagnostic & Research, Turkey) and then with povidone iodine (Adekon, Turkey), to eliminate the risk of contamination from the skin. In addition, the covers of the blood culture tubes were cleaned using 70% isopropyl alcohol in order to avoid contamination. Each blood sample (10 ml) was taken from an antecubital vein by disposable syringe (Ayset, Turkey). Blood samples were inoculated into the BACTEC bottles including aerobic and anaerobic culture media (Becton Dickinson Diagnostic Systems, Sparks, MD, USA). 

 Microbiological Analysis

The blood samples were transferred to the laboratory of the Department of Microbiology and Clinical Microbiology, Faculty of Medicine, Istanbul University within 15 minutes of collection, where all microbiological analyses were conducted. The blood culture bottles were incubated and monitored for the presence of microorganisms for 7 days in a BACTEC 9120 (Becton Dickinson Diagnostic Systems, Sparks, MD, USA) automated system. Gram staining was performed for each positive culture that was removed from the blood culture system. For the aerobic bottle, the positive blood cultures were subcultured on sheep blood agar (bioMerieux, France) and chocolate agar (bioMerieux, France) plates in an atmosphere of 5-10% CO2. For the anaerobic bottle, the same protocol was used, but the sample was subcultured on Schaedler agar (Oxoid, UK) and incubated in an anaerobic atmosphere using Gaspak pockets (Oxoid, UK). The isolated bacteria were identified using conventional methods, including colonial morphology, gram stain appearance, catalase and oxidase reactions. 

 Sensitivity to Antibiotics

The criteria of the Clinical and Laboratory Standards Institute (CLSI, Performance standards for antimicrobial susceptibility testing. 15th informational supplement. USA: 2005; M100-S15) were used for evaluation of the antimicrobial sensitivity tests.

 Statistical Analysis

The differences in patient characteristics between those with positive or negative blood cultures were analyzed using SPSS software (version 10, SPSS Inc., Chicago, IL, USA). Fisher’s exact test was used to compare gender and the Mann-Whitney U-test was used to compare number of implants, age and duration of surgery. McNemar’s test was used to compare the prevalence of bacteremia detected at baseline with second sample.

## Results

A total of 180 aerobic and anaerobic bottles (30 healthy volunteers; 3 samples, taken at baselines, after 30 minutes and 24 hours) of blood culture were processed. The characteristics of the study group are given in ( Table 1). 

In the first and third samples, no bacterium was isolated in any patient. In the second sample, seven of the 30 patients showed bacterial growth. The prevalence of bacteremia was 23% (7/30) at 30 minutes after the dental implant installation. No statistically significant differences were found between the characteristics of patients with or without bacteremia at 30 minutes after the implant surgery (p > 0.05) ( Table 2). The differences in the prevalence of bacteremia detected at baseline with second sample was found statistically significant (p= 0,016).

The isolated bacteria species were Staphylococcus epidermidis, Eubacterium spp., Corynebacterium spp. and Streptococcus viridans. ( Table 3) shows the microbiological results for patients who demonstrated a positive bacteremia. The sensitivity of the isolated bacteria to antimicrobial drugs is shown in ( Table 4). The Staphylococcus epidermidis, which was isolated in the 4th, 7th and 8th patients, was found to be resistant to penicillin. The bacteria isolated from the 8th, 23rd and 29th patients were found to be resistant to clindamycin.

## Discussion

At the beginning of the twentieth century, it was indicated that oral borne bacteria could lead to IE by forming bacteremia. Since then, the prevalence of bacteremia related to different dental applications has been investigated (4,6,9,17,18). Based on the results of experimental and clinical studies and clinical observations, different antibiotic prophylaxis are recommended for avoiding IE (14,15). The present study investigated the incidence of bacteremia after dental implant surgery and the bacterium types that cause bacteremia; their antibiotic susceptibilities; and the appropriate forms of antibiotic prophylaxis to be applied in patients at risk of IE. The results of the present study indicate that dental implant surgery can cause detectable bacteremia. 

Different rates of bacteremia were reported as a result of different applications in dentistry. Takai et al. (17) investigated the incidence of bacterium after different oral and maxillofacial surgical operations. In samples collected shortly after surgical procedures, the incidences of bacteremia were found to be 58.3% (decortication for jaw osteomyelitis), 57.9% (tooth extraction), 30.3% (orthognathic surgery), 23.1% (surgical repair of jaw fracture), 22.2% (Caldwell-Luc procedure), 18.8% (surgical reconstruction of jaw with bone graft) and 17.9% (enucleation of odontogenic cyst). Tomás et al. (19) found the prevalence of bacteremia following dental extractions to be 96.2% at 30 s, 64.2% at 15 min and 20% at 1 h after completing surgical procedure. Gürel et al. (20) found the bacteremia incidence to be 32% after the removal of a modified acrylic bonded rapid maxillary expansion appliance. Crasta et al. (9) researched the incidence of bacteremia emergence after dental flossing in individuals who were healthy in periodontal terms and in patients who had chronic periodontitis. The bacteremia incidence was found to be 41% at 30 sec. after dental flossing in the periodontally healthy patients and 40% in the patients with chronic periodontitis. At 10 minutes after the operation, the incidence decreased to 14% in the periodontally healthy patients and to 27% in the patients with chronic periodontitis. Recently, Piñeiro et al. (16) investigated the prevalence, duration and etiology of bacteremia and the efficacy of chlorhexidine digluconate as a preoperative mouthrinse against bacteremia. Patients who used 10 ml chlorhexidine digluconate mouthrinse (0.2%) before dental implant surgery had no positive blood cultures after implant insertion. In contrast to the mouthrinse group, the incidence of bacteremia in the control group was 6.7% (two of 30 patients) at 30 s after dental implant installation and 3.3% (one of 30 patients) at 15 min after completion of the suturing of the mucoperiosteal flap. No statistically significant differences were found between the baseline and post-operative percentage bacteremia. The reason why the bacteremia incidence are different between the abovementioned studies can be explained with the differences between the dental treatments, the samples were collected at different times and different techniques were used to determine bacteremia.

**Table 1:**
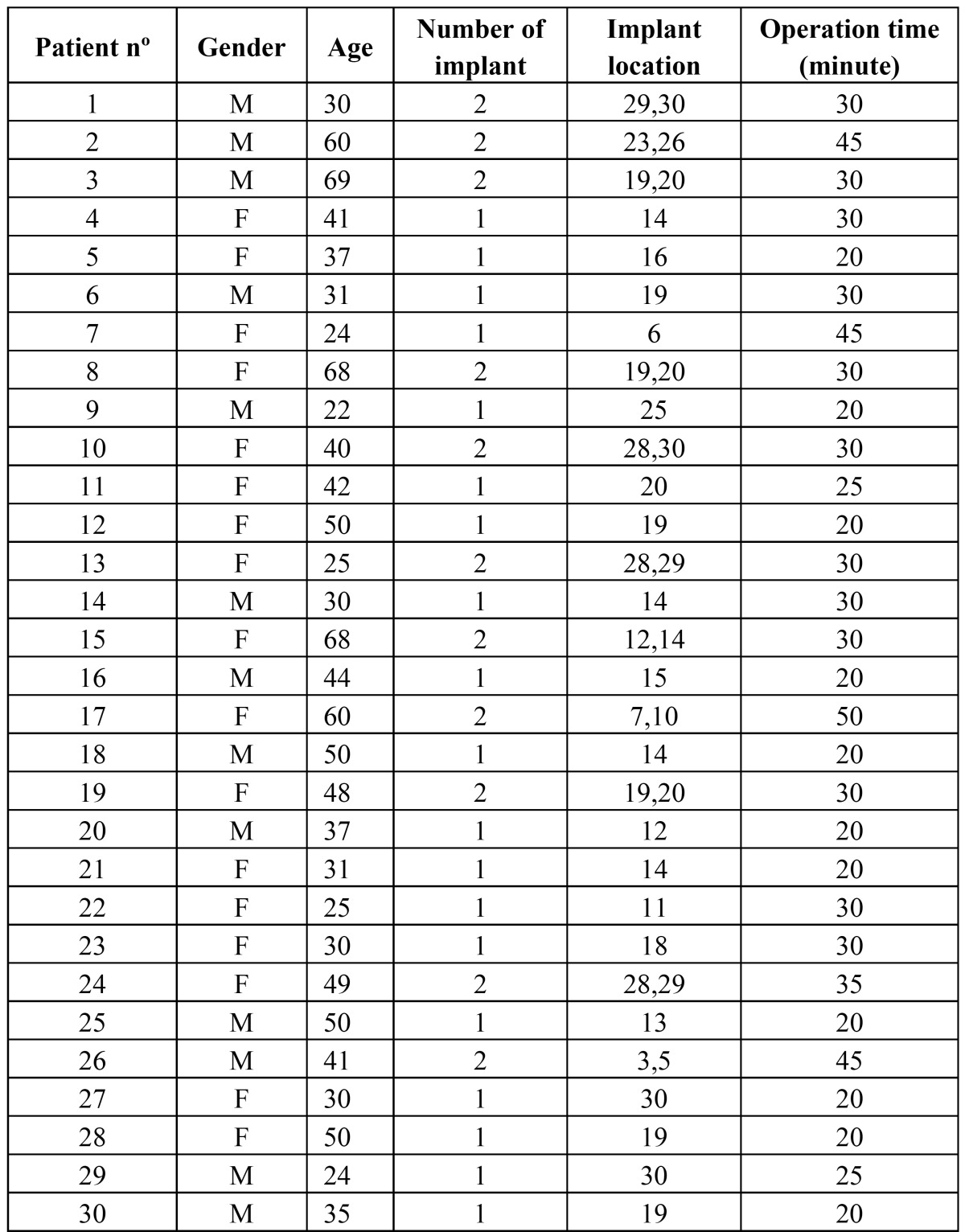
Characteristics of study group M: male, F: female. Implant location according to Universal Numbering System.

**Table 2:**
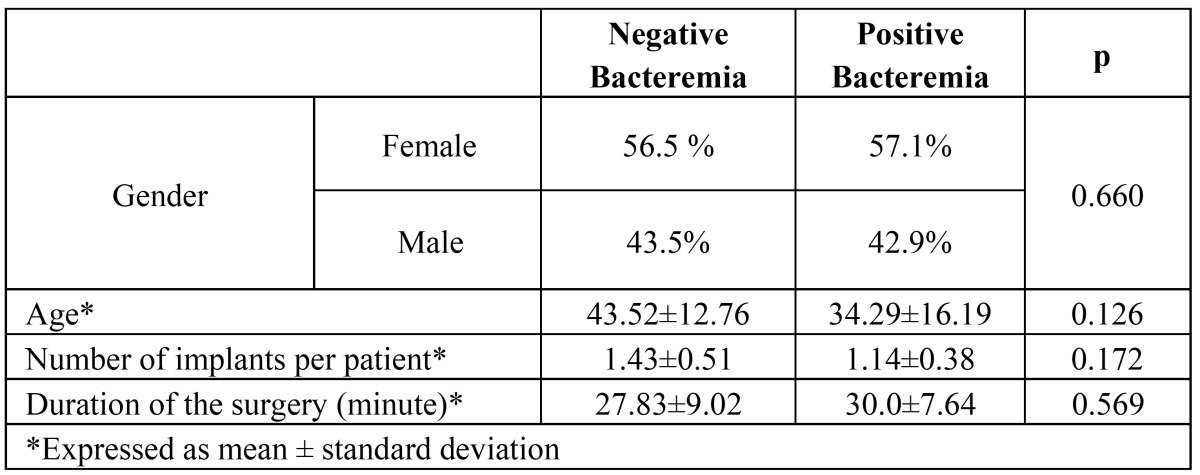
Characteristics of patients with negative or positive bacteremia at 30 minutes after dental implant surgery.

**Table 3:**
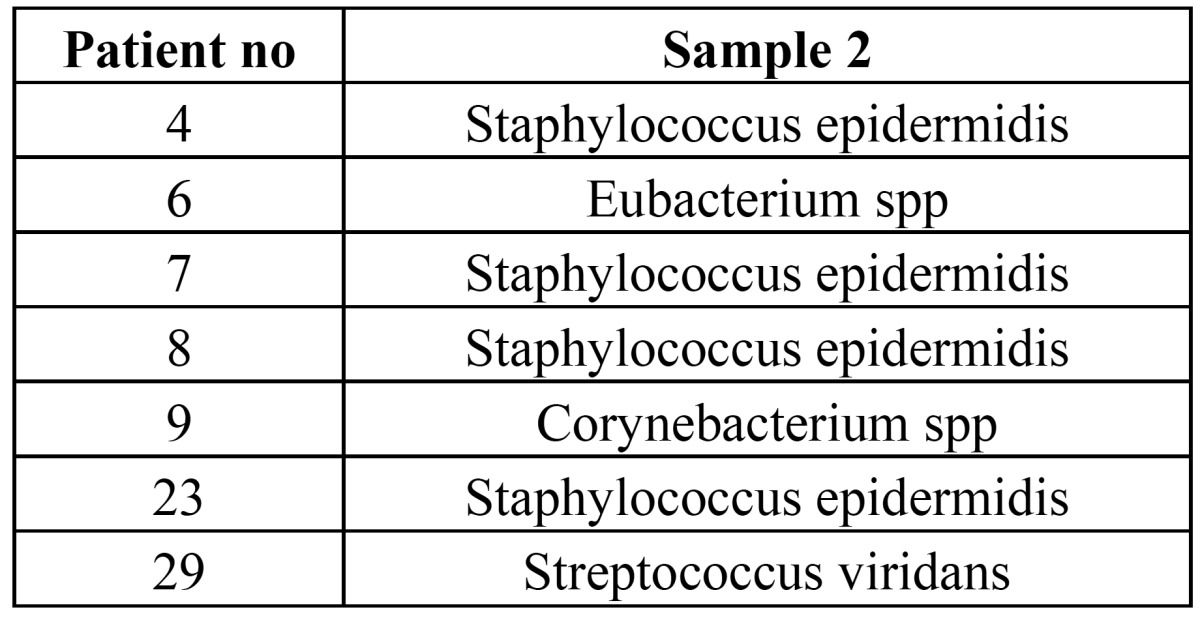
Microbiological findings of patients with positive bacteremia in Sample 2.

**Table 4:**
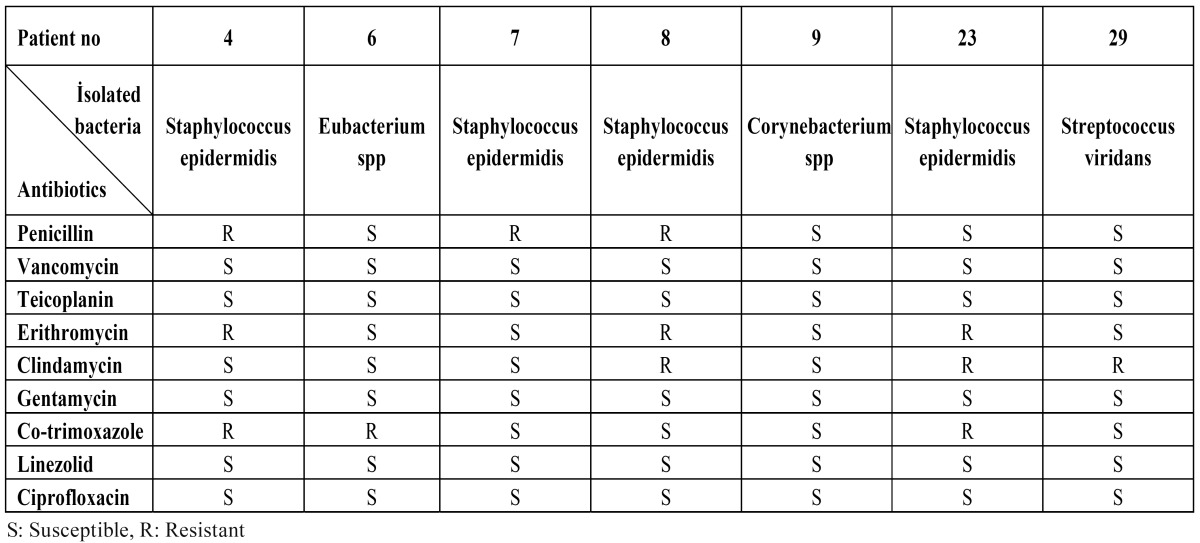
The antibiotic susceptibilities of microorganisms isolated from second blood samples of seven patients.

In many studies, Streptococcus viridans were the most frequently isolated bacteria in positive blood cultures (4,9,16,17,19,20). In a study by Piñeiro et al. (16), the bacteria isolated after dental implant surgery was Streptococcus viridans and Neisseria cinerea. In the present study, Staphylococcus epidermidis was found to be the most common bacteria isolated (4/7= 57.1%) from the second sample. In the other three patients, Eubacterium spp., Corynebacterium spp. and Streptococcus viridans were isolated. Staphylococcus spp. is considered microorganisms of the skin. Although skin disinfection protocols were used in this study, contamination might have occurred. Roberts et al. (21) found Staphylococcus spp. in 9% of positive blood cultures. They reported that up to 6% of these positive results could be attributed to contamination. If we take account of Roberts et al’s study, bacteremia incidence would be lower in our study. 

Since it was expected that oral borne bacteremia would be transient, early studies investigated the presence of bacteremia at times varying between 30 seconds and hour after the operation (2,4,9,16). Contrary to these expectations, the incidence of bacteremia was found to be 8% at 24 hours after the third molar tooth extraction (22). As there is no adequate data regarding the period within which bacteremia may emerge after an implant surgery, blood samples were collected after 24 hours in the present study and the incidence of bacteremia was found 0%. 

In detection of bacteremia, the lysisfiltration method, polymerase chain reaction (PCR) and BACTEC growth bottles can be used. The lysisfiltration technique takes longer, and thus is less appropriate for clinical use (18). The PCR technique is very sensitive, but it does not discriminate between live and dead bacteria (4). The BACTEC blood culture system is a fully-automated microbiology growth and detection system, designed to detect microbial growth from blood specimens. Due to the above-mentioned disadvantages of the other systems and the widespread use and established reliability of BACTEC, the BACTEC automated system was used in the present study (4,5,9,16,19). 

There is no conclusive evidence within the literature, on whether or not oral health status influences bacteremia. While some au-thors indicate that the bacteremia risk increases in individuals who have periodontal or odontogenic infection (17,18), other au-thors indicate that this is not a risk factor (9,19). In the present study, radiological and clinical examinations were made before the dental implant surgery, and patients who had good oral hygiene and did not have odontogenic infection were included in the study. This method was selected in order to eliminate the influence of periodontal and odontogenic infections on the results.

In this study, the group was composed of individuals with single tooth loss or partial edentulism. Therefore, data about the bacteremia formation in people with total edentulism could not be acquired. Since people with total edentulism do not have periodontal cavities or dental plaque, they are expected to experience bacteremia less frequently than patients who have teeth (17). In recent years, implant applications in totally edentulous older patients have increased (23). Furthermore, these older patients are at risk of cardiac abnormalities and immune system deficiencies (24). As a result, it will be reasonable to conduct further studies on the risk of bacteremia in totally edentulous patients who undergo implant surgery. 

In dental implant surgery mucoperiosteal flap is necessarily elevated in edentulous areas. Therefore it has been demonstrated that the use of the mucoperiosteal flap procedure in dental implant surgery does not cause significant bacteremia (16). Also there is inadequate data on which individuals are at risk of developing IE following dental implant surgery, the incidence of bacteremia and antibiotic susceptibility. Therefore, clinicians apply prophylaxis, either empirically, or in accordance with the guidance of organizations such as the AHA and BSAC. One of the most important problems that may be encountered while using the AHA and BSAC guidelines is resistance to antibiotics. Nishi et al. (25) found that Streptococcus viridans resistance was 61% in oral flora in children with a high risk of IE. Groppo et al. (26) examined the antibiotic susceptibilities of Staphylococcus aureus and viridans streptococci, which appear in skin and saliva, among patients with a high risk of IE. Of the Staphylococcus aureus strains, 50% were resistant to ampicillin, 53.3% to amoxicillin, 60.0% to penicillin G, 13.3% to amoxicillin/clavulanate, 20.0% to azithromycin, 27.6% to clarithromycin, 23.3% to erythromycin, 3.3% to cefazolin, and 6.7% to clindamycin. Of the streptococci strains examined, 16.7% were resistant to ampicillin, 16.7% to amoxicillin, 23.3% to azithromycin, 23.3% to clarithromycin, 30.0% to erythromycin, 13.3% to cefazolin, 26.7% to clindamycin, 16.7% to penicillin G, and 3.3% to amoxicillin/clavulanate. 

In the present study, the Staphylococcus epidermidis strains that were isolated from the 4th, 7th and 8th patients were found to be resistant to penicillin. Streptococcus viridans were found to be resistant to clindamycin. For ethical reasons, the present study included only patients whose general health was good and who did not have an increased risk of IE. However, if the patients had been at risk of IE and even if the prophylaxis methods suggested by AHA and BSAC had been implemented, it is possible that IE might have developed due to resistance to antibiotics.

## Conclusion

The results of the present study indicate that bacteremia may occur following dental implant surgery. It is necessary to conduct further, larger-scale studies to investigate the incidence of bacteremia following dental implant applications. There is no consensus on which specific patient population is at risk of developing IE following dental implant surgery. Furthermore, it is unclear which antibiotic prophylaxis should be selected, and AHA and BSAC guidelines might be insufficient for the prevention of IE. As a result, the use of blood cultures and antibiograms may be suggested for patients who are at increased IE risk in implant surgery.
